# The Evaluation of Indicators Used to Assess the Suitability of Agricultural Waste for Fermentation

**DOI:** 10.3390/ijerph16111889

**Published:** 2019-05-28

**Authors:** Monika Suchowska-Kisielewicz, Andrzej Jędrczak

**Affiliations:** Institute of Environmental Engineering, The University of Zielona Góra, 65-417 Zielona Góra, Poland; a.jedrczak@iis.uz.zgora.pl

**Keywords:** biodegradation of agricultural waste, fermentation of agricultural waste, respirometric activity (AT4), the biochemical methane potential (BMP)

## Abstract

To ensure high fermentation efficiency, it is necessary to assess the biodegradability of a substrate. These parameters are most often determined on the basis of the amount of loss on ignition and total organic carbon. We are more and more often using chemical indices. However, these indices do not provide information on how much an organic substance is susceptible to biodegradation. The actual assessment of the content of easily biodegradable matter in substrates that are used for fermentation should be performed on the basis of aerobic (AT4) and anaerobic tests (BMP), which require specialised equipment and are time consuming. The AT4 index is being more and more frequently adopted for the analysis of substrates that are used in the fermentation process, because AT4 takes a much shorter time than BMP and provides information on the biodegradability of substrates. The aim of the article is to answer the question of whether the AT4 parameter can be used to assess the suitability of the substrate from the agricultural sector for the fermentation process. The results show that the AT4 index could be used instead of the BMP parameter.

## 1. Introduction

Agricultural waste due to a high proportion of organic matter is a valuable substrate that is used in agricultural biogas plants. The effectiveness of fermentation of these substrates depends on the content of the organic matter susceptible to biodegradation. The fermentation of agricultural waste is a time tested and widely used waste management method. The main advantages of fermentation include the production of electricity and heat from biogas or biomethane, which are sources of renewable energy. An additional benefit of fermentation is the production of fertilisers and a reduction in greenhouse-gas emissions and odours [1].

Biogas can be produced from a wide range of waste. Practically any organic substance, if free of inhibitors, can be used as a substrate to produce methane. Agricultural-biogas plants use waste from the agro-food industry, and are both easily biodegradable (such as fruit waste, potato peelings, waste from the production of oil, cheese, brewer’s grain, pig manure, cattle manure, and chicken manure) as well as lignocellulose waste from horticulture (leaves, stalks), forestry (branches, logs) or waste from energy crops (e.g., maize, rape, sugar beet). The high efficiency of methane production from waste is achieved in the process of its co-fermentation (the fermentation of a mixture of two or several components), which makes it possible to optimise the composition of substrates, in particular dry-matter content, organic dry-matter content, the C/N ratio, and inhibitor concentration [2].

The benefits resulting from the co fermentation of waste include an increase in the daily methane yield per bioreactor unit volume, an increase in the methane content in biogas, and an improvement in the fertilising properties of digestate sludge [3].

A favoured waste substrate for Polish biogas plants is chicken manure. According to the literature data, the content of organic dry mass (DM) in chicken manure ranges from 63% to 80% DM, biogas production equals 250–450 m^3^/Mg DOM (dry organic mass), and the substrate enables achieving a 60% (volume) concentration of methane in biogas. [2].

Poland is currently the leader in poultry production in the European Union. According to the National Poultry Council, the production of poultry meat in Poland in 2017 amounted to approximately three million tonnes.

The chicken and other livestock breeders are currently finding it difficult to meet the strict legal requirements. The obligation to develop at least 70% liquid manure and slurry produced during the rearing of animals on agricultural land which belongs to them, and where they grow crops, is particularly difficult to fulfil for the breeders [4]. This forces the breeders to have large plots of land. The annual nitrogen dose in the natural fertiliser deposited in the soil may not exceed 170 kgN/ha. This means that the acceptable population of chickens per 1 ha of land is around 580.

As a result of the fermentation of chicken manure, not only is biogas obtained, which can be used for energy purposes, but its fertilising properties are improved as well. Natural fertilisers (liquid manure, manure) are in most cases currently collected in lagoons or on manure plates, which leads to the emission of carbon dioxide and methane. One of the methods for reducing emissions is the utilisation of natural fertilisers in agricultural biogas plants. In addition to the emission of greenhouse gases in the case of natural fertilisers, there is also a significant emission of nitrogen at every stage of their production (collection, storage, field application).

However, the use of chicken manure as a substrate in agricultural biogas plants creates operational problems. This is mainly due to the high concentrations of ammonium nitrogen, and the unfavourable ratio of organic carbon to nitrogen (C/N), ranging from 2:1 to 14:1 [3]. The optimal C/N ratio for the methane fermentation process is from 20:1 to 30:1 [5]. Therefore, properly running the methane fermentation of chicken manure requires balancing the C/N ratio by introducing an appropriate amount of co-substrates that are rich in organic carbon [6]. Co-substrates might be greenhouse waste (tomato and cucumber blades), agricultural waste (peel, pulp, molasses), biomass, including energy crops (maize silage, grasses), organic fraction of municipal waste, sewage sludge, etc.

To ensure high co-fermentation efficiency, it is necessary to assess the biodegradability of the substrate entering the fermentation chamber, and the degree of its fermentation [1].

These parameters are most-often determined on the basis of the amount of loss on ignition (VS) and the total organic carbon content in the organic substrate (TOC). We are more and more often using the chemical indices that, until now, have been used to assess the content of organic matter in wastewater. These include the chemical oxygen demand (COD), which is an index that determines the amount of oxygen obtained from oxidants needed for the oxidation of organic compounds as well as some inorganic elements (sulphates, sulphides, iron II) and the biochemical oxygen demand (BOD), which is an index showing the amount of oxygen required for the oxidation of organic compounds by microorganisms (aerobic bacteria).

These indices do not provide information on how much of the organic substance present in the fermented substrate is biodegradable. However, since they are easy to obtain, they are widely used for determining substrate characteristics and the efficiency of the fermentation process. However, due to the limited amount of information available on the degree of biodegradation of organic matter, they might be insufficient to assess the actual efficiency of the process [1,7,8].

The actual content of easily biodegradable matter in substrates subjected to fermentation can be found through aerobic and anaerobic tests, which require time and specialised equipment. The time needed for these anaerobic test ranges from 21 to 100 days.

- BMP—the biochemical methane potential test—duration up to 100 days,
- GS21—the total gas production test—duration 21 days,
- GB21—the assessment of biogas production—duration 21 days,

and the aerobic (respiratory) tests, which last from four to 100 days:

- AT4—the static respiratory test—duration four days,
- DR4 and DR 100—the dynamic respiratory test—duration four and 100 days, respectively.

The AT4 index is gaining in popularity as a method for the analysis of substrates used in the fermentation process, because AT4 is less time consuming than BMP, it provides information on the biodegradability of substrates, and has a high correlation with anaerobic tests. An important advantage of this test is that it can be used for a wide range of substrates [9].

Numerous publications present correlations between particular indices, which in most cases have high values of R2 coefficients [10,11,12]. However, they mainly involve municipal waste, and their aim is to assess the degree of stabilisation of waste discharged into landfills, as well as predict how much biogas will be produced from stored stabilised waste. There has not been much research on the subject of the biochemical methane potential of chicken manure, and the correlation between its values and AT4 indices, or the physical and chemical indices of waste from the agro-food sector, i.e.; substrates widely used in the fermentation process.

The aim of this study is to present the correlation between VS, COD, AT4, and BMP indicators determined for chicken manure, maize silage, grass and tomato stalks, and their mixtures, and to answer the question of whether the AT4 parameter can be used to assess the suitability for the fermentation process of substrate from the agricultural sector.

## 2. Materials and Methods

The research involved the following waste products, comminuted to the size of <20 mm: chicken manure (C), maize silage (M), grass (G), and tomato stalks (T), and mixtures of chicken manure with other waste in different proportions.

The following mixtures were tested in the study.

- Maize silage + chicken manure
  - 80% maize silage + 20% chicken manure
  - 70% maize silage + 30% chicken manure
  - 60% maize silage + 40% chicken manure
  - 40% maize silage + 60% chicken manure
  - 30% maize silage + 70% chicken manure
- Tomato stalks + chicken manure
  - tomato stalks 90% + 10% chicken manure
  - tomato stalks 80% + 20% chicken manure
  - tomato stalks 70% + 30% chicken manure
  - tomato stalks 60% + 40% chicken manure
  - tomato stalks 40% + 60% chicken manure
- Grass + chicken manure,
  - 80% grass + 20% chicken manure
  - 40% grass + 60% chicken manure
  - 30% grass + 70% chicken manure
  - 10% grass + 90% chicken manure

The scope of the study on waste and its mixtures included:

- the determination of the content of organic substance expressed as VS and COD
- the determination of biodegradability under aerobic and anaerobic conditions based on AT4 and BMP tests.

VS and COD were determined in accordance with the APHA methodology.

**Figure 1:**
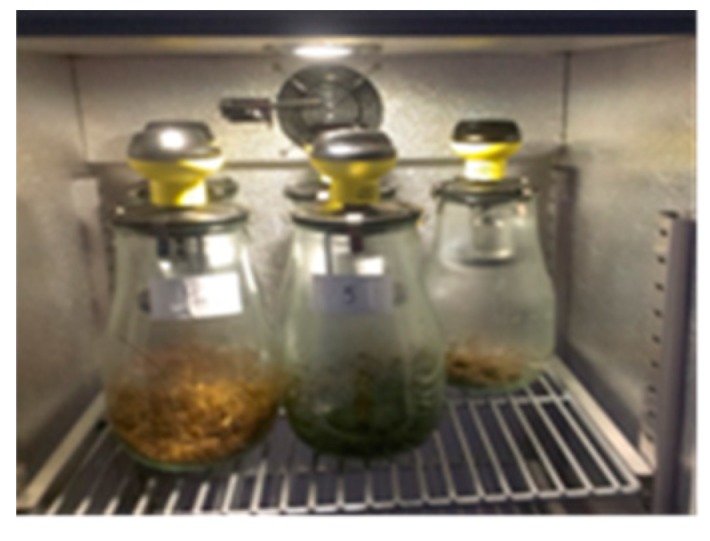
Test stand—the AT4 test.

The AT4 test was carried out using the static method and an Oxi Top B 6M-2.5 device (WTW, Weilheim in Oberbayern, Germany) [13,14,15]. Waste samples were reduced in size to dimensions below 20 mm, and brought to a moisture level of 40–50%. Microbial activity was measured for four days, counting from the end of the adaptation phase. It was assumed that this phase ended when the average oxygen demand over a period of three hours reached 25% of the three-hour average value, which had occurred during the maximum increase in oxygen consumption during the first four days. The mass of oxygen consumed during the lag phase was not included in the mass of oxygen consumed during the whole test (lag phase + four days). The mass of oxygen from the lag phase cannot exceed 10% of the total demand for oxygen during the first four days. The tests were carried out at a constant temperature of 20 °C in a thermostatic cabinet (Figure 1).

**Figure 2:**
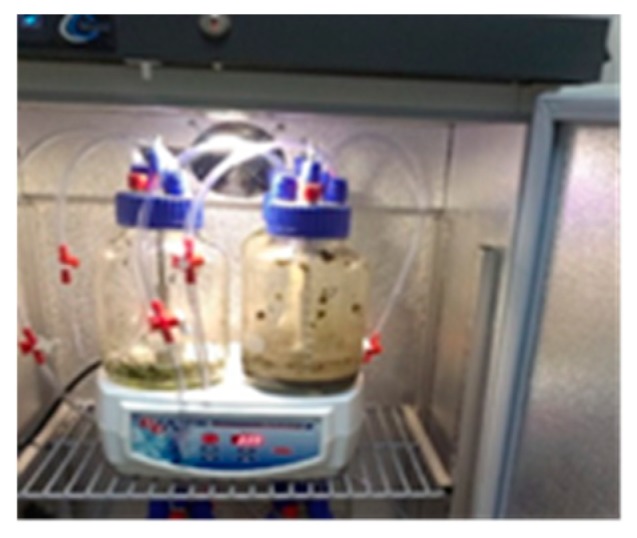
Test stand—the BMP test.

The research on the BMP of substrates was carried out in anaerobic reactors with a capacity of 2.5 dm^3^ [13,16]. The process was carried out for 21 to 30 days at a temperature of 37 °C (Figure 2). The biogas produced was collected from the reactors using a 300-ml syringe. The content of CH_4_, CO_2_, O_2_, NH3, and H2S in the biogas was measured with a GEOTECH BIOGAS 500 analyser (Geotechnical Instruments Ltd, Warwickshire, England). At the beginning of the process, the volume of the biogas produced and its composition were measured daily, and then depending on the volume of biogas production. In order to maintain the optimum conditions for decomposition, the contents of the reactors were mixed mechanically. The amount of substrate introduced into the reactor was determined. It was assumed that at least 395 ml of CH_4_ would be produced from 1 g of COD. The amount of inoculum was determined based on the VS of the substrate. It was assumed that there was 1 g VS of inoculum per 1 g VS of the substrate [11]. The tests were performed in triplicate.

## 3. Results and Discussion

### 3.1. The Characteristics of Substrates

The content of organic matter in the waste, and its susceptibility to biological decomposition, are shown in Table 1.

As expected, in the group of the substrates analysed, the highest content of organic matter expressed by the VS and COD indices, and the highest susceptibility to biological degradation, were found in maize silage, and the lowest values were found in chicken manure. The values of the methane biochemical potential and the oxygen respiration index determined for maize silage amounted to 320 dm^3^/kg of DM and 200 g/kg of DM respectively, and were about 74% and 83% higher than the values determined for chicken manure. The values of the VS and COD indices for these substrates were higher by 35% and 63%, respectively (Table 1). 

The values of the biochemical methane potential and the oxygen respiration index for tomato stalks were only about 10% higher than the values determined for chicken manure. The values determined for grass were higher by 29% and 49%.

As expected, in most cases, the addition of other substrates to the chicken manure led to an increase in the content of VS and COD, and consequently to an increase in the BMP and AT4 indicators.

**Figure 3:**
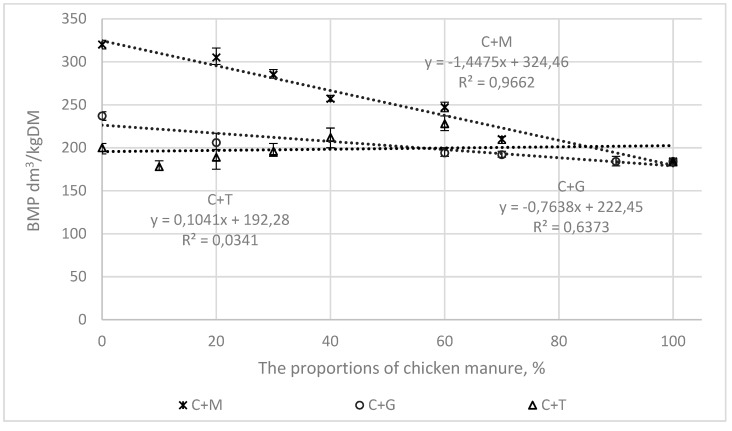
The correlations between the BMP and the proportions of chicken manure in the mixtures with co-substrates.

**Figure 4:**
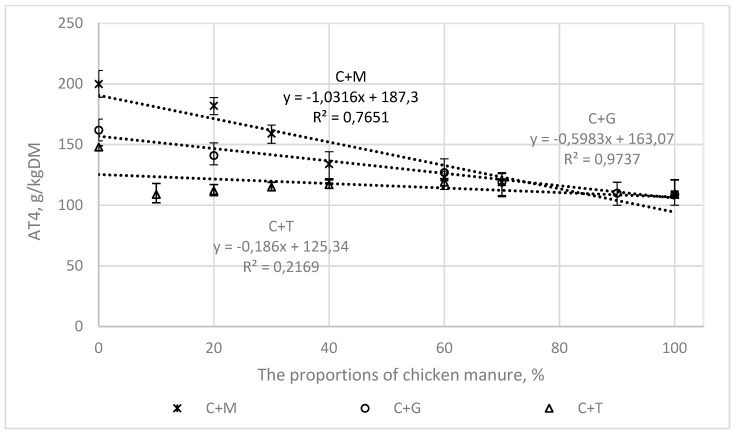
The correlations between AT4 and the proportions of chicken manure in the mixtures with co-substrates.

**Figure 5:**
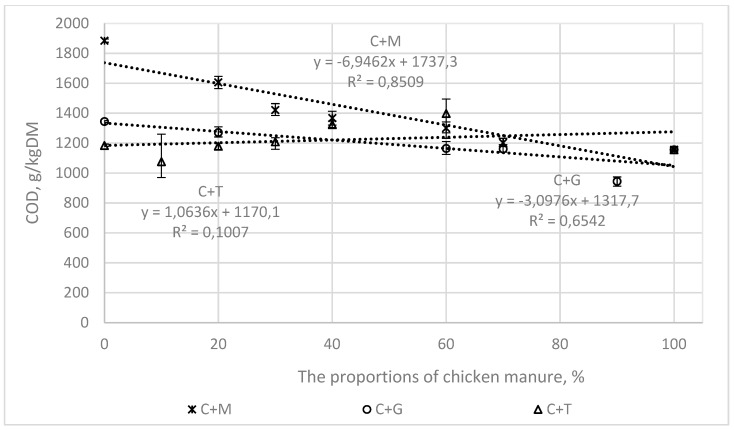
The correlations between COD and the proportions of chicken manure in the mixtures with co-substrates.

**Figure 6:**
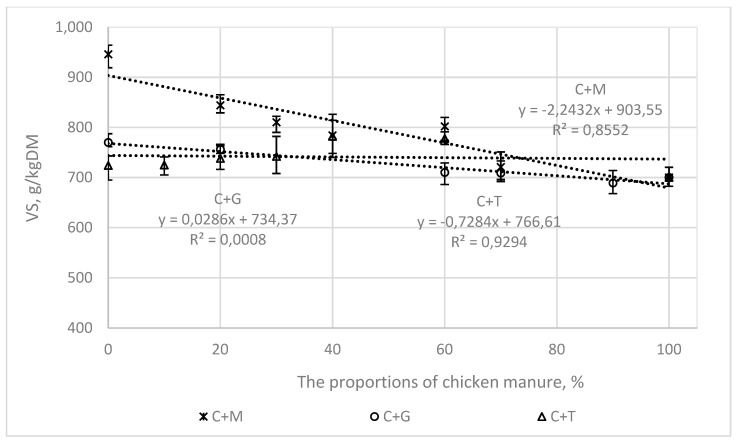
The correlations between VS and the proportions of chicken manure in the mixtures with co-substrates.

Figure 3, Figure 4, Figure 5 and Figure 6 present the correlations between the BMP, AT4, VS, and COD values for the mixtures and the proportions of chicken manure in them, as well as the corresponding ranges from the minimum to the maximum values. 

In the mixtures of chicken manure with maize silage (C + M) and grass (C + G), the correlations were linear, with the following values of the coefficient of determination (R^2^).

- 0.97 and 0.63, for BMP (Figure 3)
- 0.77 and 97 for AT4 (Figure 4)
- 0.85 and 0.65 for COD (Figure 5)
- 0.86 and 0.93 for VS (Figure 6).

For tomato stalks, the correlations between the studied indicators (BMP, AT4, COD, and VS) and the proportions of chicken manure were not shown to be statistically significant.

### 3.2. The Correlations between the AT4, COD, and VS Indices and the BMP

**Figure 7:**
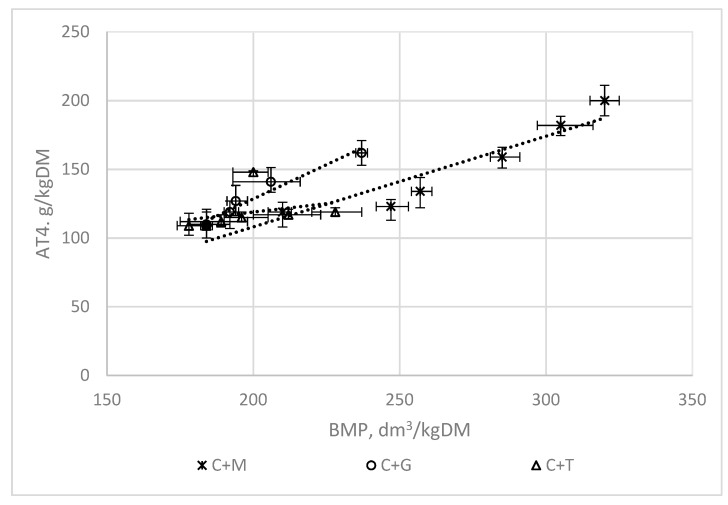
The correlations between AT4 and the BMP.

**Figure 8:**
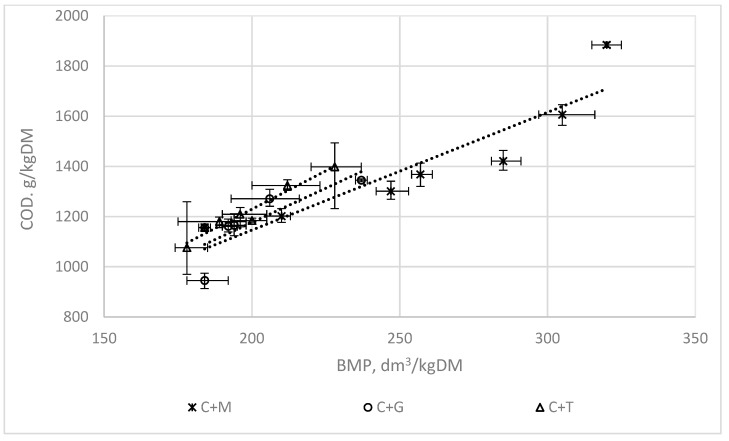
The correlations between the COD index and the BMP.

**Figure 9:**
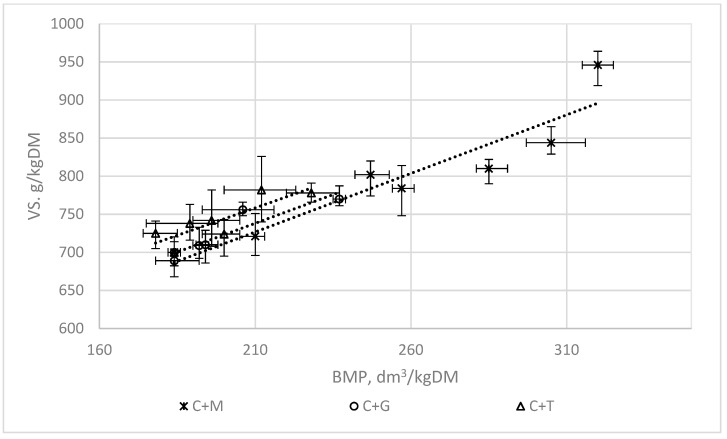
The correlations between the VS index and the BMP.

Figure 7, Figure 8 and Figure 9 present the correlations between the AT4, COD, VS, and BMP indices for mixtures of chicken manure with maize silage (C + M), with grass (C + G), and with tomato stalks (C + T). The means of the triplicates are presented jointly with the standard deviations.

Table 2 presents the linear functions describing the correlations between these indices and the coefficients of determination R^2^.

The functions shown in Table 2 have coefficients of determination R^2^ ranging from 0.53 to 0.95 (Figure 7, Figure 8 and Figure 9).

The highest correlations were obtained for the functions describing the correlations between the indices used to assess the biodegradation of waste AT4 and the BMP (R^2^ = 0.88–0.95). The results confirm the data found in the literature indicating that the AT4 index could be used instead of the BMP parameter [17]. The main advantage of using the AT4 index is the significant reduction in the time needed to evaluate the usefulness of the substrates or their mixtures for fermentation, and to check their fermentation efficiency.

The correlations between the VS and COD indices and the BMP were not so favourable.

The lowest coefficients of determination for these correlations, amounting to about 0.50, were found for the mixtures of chicken manure with tomato stalks. Tomato stalks can be regarded as a moderately biodegradable substrate, which has a higher content of lignocellulose components than maize silage or grass [16]. 

The biodegradation of waste rich in liglinocellulose compounds is usually more time consuming due to the complex structure of the lignin and other cell-well polysaccharides, which results in lower methane production compared to the production of carbohydrates, fats, or proteins [16,18,19]. For this type of waste, the BOD_5_/COD quotient should be a more useful index for assessing biodegradation. Further tests will be carried out to confirm this.

On the basis of this study, it can be concluded that the assessment of the suitability of substrates for the fermentation process should be carried out not on the basis of the VS and COD content, but mainly on the basis of the AT4 or BMP indicators. However, because the BMP test is time consuming (taking anywhere from 21 to 100 days to complete, depending on the substrate in question), AT4 testing is now increasingly being used in practice [20,21,22]

This is illustrated by the results presented in this article, which showed that the correlation between VS, COD, and BMP was highest for the mixtures of C + M and C + G (easily biodegradable mixtures), while for the mixture of C + T (which is only moderately biodegradable), it was significantly lower.

## 4. Conclusions

The paper presents an analysis for checking whether it is possible to use the VS, COD, and AT4 indices for assessing the susceptibility of waste from the agro-food sector to biodegradation in anaerobic conditions.

The results led to the following conclusions.

1. The AT4 index is a parameter that makes it possible to assess the suitability of a substrate for fermentation, and to monitor the fermentation effectiveness, replacing other indicators such as BMP, VS, and COD.
2. The VS and COD indices can be used to assess the biodegradation susceptibility of easily and moderately biodegradable substrates, but they do not give reliable information on the content of readily decomposable organic substances.
3. Further tests will be carried out to prove the usefulness of the BOD_5_/COD quotient as a biodegradation index for waste with medium and low susceptibility to decomposition.

## Figures and Tables

**Table 1:** The physico-chemical composition of the substrates and their susceptibility to biological decomposition. AT4: aerobic, BMP: biochemical methane potential, COD: chemical oxygen demand.

No.	Percentage of Substrates	VS g/kg DM	SD *	COD g/kgvDM	SD *	BMP dm^3^/kg DM	SD *	AT4 g/kg DM	SD *
1	Chicken manure	700	19.14	1156	13.20	184	2.08	109	10.69
2	Maize silage	946	23.81	1884	10.60	320	5.03	200	11.02
3	Grass	770	15.01	1345	6.03	237	2.08	162	9.87
4	Tomato stalks	760	25.51	1184	8.33	200	6.24	120	2.08
5	80% maize silage, 20% manure	844	18.73	1606	41.04	305	9.71	182	7.02
6	70% maize silage, 30% manure	810	17.44	1421	39.96	285	5.13	159	7.55
7	60% maize silage, 40% manure	784	33.41	1368	46.57	257	3.79	134	11.14
8	40% maize silage, 60% manure	802	24.58	1301	36.66	247	5.69	123	8.66
9	30% maize silage, 70% manure	721	27.84	1202	27.84	210	4.16	119	9.64
10	80% grass, 20% manure	756	9.17	1271	34.70	206	11.93	141	9.29
11	40% grass, 60% manure	710	21.93	1165	43.71	194	3.61	127	11.02
12	30% grass, 70% manure	709	21.38	1161	26.21	192	2.52	119	10.58
13	10% grass, 90% manure	689	23.26	945	30.61	184	7.21	110	9.54
15	80% tomato stalks, 20% chicken manure	738	23.64	1180	159.14	189	12.29	112	8.33
16	70% tomato stalks, 30% chicken manure	742	37.36	1183	21.63	196	8.14	115	4.73
17	60% tomato stalks, 40% chicken manure	782	43.03	1330	43.35	202	11.59	117	3.79
18	40% tomato stalks, 60% chicken manure	778	12.53	1329	21.66	208	8.54	119	3.79

* SD—standard deviation.

**Table 2:** The functions describing the correlations between the AT4, COD, and VS indices, and the BMP.

Mixture	Function	Coefficient of Determination R^2^
Relationship AT4 between BMP		
Chicken manure + maize silage	AT4 = 0.6598 BMP + 23.858	0.90
Chicken manure + grass	AT4 = 0.9953 BMP + 70.560	0.95
Chicken manure + tomato stalks	AT4 = 0.4001 BMP + 36.861	0.88
Relationship COD between BMP		
Chicken manure + maize silage	COD = 4.692 BMP + 207.84	0.84
Chicken manure + grass	COD = 5.4665 BMP + 83.275	0.66
Chicken manure +tomato stalks	COD = 5.3115 BMP + 147.19	0.53
Relationship VS between BMP		
Chicken manure + maize silage	VS = 1.5374 BMP + 403.92	0.87
Chicken manure + grass	VS = 1.5068 BMP + 421.72	0.86
Chicken manure + tomato stalks	VS = 2.0963 BMP + 334.90	0.57
